# Association of Continuity of General Practitioner Care with Utilisation of General Practitioner and Specialist Services in China: A Mixed-Method Study

**DOI:** 10.3390/healthcare9091206

**Published:** 2021-09-13

**Authors:** Tao Zhang, Xiaohe Wang

**Affiliations:** Department of Health Management and Policy, School of Public Health, Hangzhou Normal University, No. 2318, Yuhangtang Rd., Hangzhou 311121, China; lucky1230405@163.com

**Keywords:** continuity of GPs care, intention to visit GPs, GP and specialist visitations, China

## Abstract

*Background*: Continuity of general practitioner (GP) care, widely known as the core value of high-quality patient care, has a positive association with health outcomes. Evidence about the relationship between continuity and health service utilisation has so far been lacking in China. This study aimed to analyse the association of continuity of GP care with utilisation of general practitioner and specialist services in China. *Method*: A cross-sectional mixed methods study was conducted in 10 urban communities in Hangzhou. Quantitative data were collected from a random sample of 624 residents adopting the self-developed questionnaire. Measurement of continuity of GP care included informational continuity (IC), managerial continuity (MC) and relational continuity (RC). With adjustment for characteristics of residents, multivariate regression models were established to examine the association of continuity of GP care with the intention to visit GP, frequency of GP and specialist visitations. Qualitative data were collected from 26 respondents using an in-depth interview, and thematic content analysis for qualitative data was conducted. *Results*: Quantitative analysis showed that the IC was positively associated with the intention to visit GP and frequency of GP visitations. Those people who gave a high rating for RC also used GP services more frequently than their counterparts. MC was negatively associated with frequency of specialist visitations. Qualitative analysis indicated that service capabilities, doctor–patient interaction and time provision were regarded as three important reasons why patients chose GPs or specialists. *Conclusions*: Overall, high IC and RC are independently associated with more GP service utilisation, but a high MC might reduce specialist visitations. Continuity of GP care should be highlighted in designing a Chinese GP system.

## 1. Introduction

In 2009, the Chinese government launched a new round of healthcare system reform with the goal of establishing a basic healthcare system covering urban and rural areas and providing affordable and equitable basic healthcare for all [1]. In this round of medical and health system reform, primary healthcare providers are paid increasing attention. In the Chinese health system, primary healthcare doctors and specialists are given different functions and positions. Primary healthcare providers in community health centres (CHCs), as health gatekeepers, provide common disease management, immunisation and primary community health prevention, rehabilitation and family planning and referral to hospitals for complex problems. Specialists are supposed to pay more attention to patients with serious illnesses than general practitioners (GPs). However, a serious shortage of health resources exists in primary care institutions in China, and primary healthcare professionals in China have low levels of training, commonly do not have certifications and are paid low wages and minimal benefits. As a result, services delivered by primary care institutions are of poor quality [2]. Consumers commonly seek medical attention from specialists for minor illness or chronic conditions. To strengthen primary healthcare, China’s State Council announced the policy to propose the establishment of a GP system in 2011 with an aim of shifting patients away from overcrowded hospitals and easing the strain off the current healthcare system [3].

In this mode, residents can choose their GPs voluntarily from local primary care centres, such as community health service institutions in urban areas. A resident can only sign a health service contract with one GP, while one GP can sign as many contracts with residents as is appropriate based on their own capacity, or that of the GP team. The contracts remain in force for at least 1 year. If the resident is not satisfied with the GP, he/she has the right to choose another one for the next year. After signing, the residents are encouraged to visit their signed GPs when first feeling discomfort (except for emergencies) and are referred to a specialist at secondary or tertiary hospitals only when the GP believes it is necessary. After treatment in secondary or tertiary hospitals, patients would be referred to their GP to receive follow-up treatment or rehabilitation [4]. For the continuous and effective operation of this system, some supporting measures are necessary. Such measures include providing some basic medical and public health services for signed residents free of charge or at discounted prices and giving priority to referral patients for treatment at secondary or tertiary hospitals.

Although encouraging signs were found after these efforts, severe shortage of the health workforce in the primary care setting with less than two GPs per 10,000 people is a critical problem [5]. To address this problem, many strategies have been adopted to train and recruit GPs. For example, the new ‘5 + 3′ model of medical education was introduced to shorten the training cycle of GPs [6]. Unfortunately, patients still have no confidence in the quality of GP services and prefer to choose specialists in tertiary hospitals for treatment, even for minor diseases [7]. In the past few decades, the great disparity in the distribution of patients between primary care setting and tertiary hospitals has caused an excessive increase in health expenses in China [8]. Currently, the number of consultations in CHCs has increased to a certain extent with the implementation of the GP system [9,10], but competency of GPs and poor quality of GP services remain serious problems criticised by patients in China [2].

Continuity of care (COC) is considered a vital characteristic of general medicine, and the Institute of Medicine holds that COC, defined as an ongoing partnership between patients and physicians, is a core component of high-quality primary healthcare services [11]. According to the conceptual framework from Reid et al. [12], COC can be classified into three types: informational continuity (IC), management continuity (MC) and relational continuity (RC). RC refers to the patient’s opinion on an ongoing therapeutic relationship with one or more providers that connects care over time. IC is defined as the patient’s perception of the availability and use of information on past events and personal circumstances by the physician, whereas MC refers to the patient’s view about the provision of separate types of healthcare in ways that they complement each other and are connected in a coherent way. Those three types are closely related and may vary in importance depending on patients’ characteristics, or the process of care.

The advantages of COC have been documented extensively in developed countries. The relationship between COC and outcomes is particularly well supported, such as patient satisfaction with healthcare, hospitalisation rates, emergency department visits, mortality and healthcare cost [13,14,15,16]. However, some gaps still exist. Firstly, most studies mainly focused on the association between a single aspect of COC and related outcomes, such as RC [17], and rarely involved comprehensive COC. Secondly, the relationship between COC and seeking health service behaviour is little known because previous studies focused on the effect of COC on patient outcomes. Thirdly, few studies focused on the continuity of GP care in China. No evidence was provided to demonstrate that continuity of primary care is significantly associated with health service utilisation in China’s healthcare system.

In the background of the GP-based primary healthcare system in China, the providers are proposed to build a stable and continuous contractual service relationship with residents and provide continuous health services [18]. Thus, the importance of COC in the GP system is particularly highlighted to change the patient’s preference for specialists. However, whether continuity of GP care can change the patient’s behaviour in choosing a doctor when sick is unknown. In other words, patients are willing to continuously utilise primary health services and voluntarily reduce the frequency of specialist visitations in secondary and tertiary hospitals. Evidence to verify this relationship is lacking. In this context, this study aimed to test the association of three types of continuity of GPs (IC, MC and RC) with intention to visit GPs, frequency of GPs and specialist visitations using data from Hangzhou, China.

## 2. Materials and Methods

### 2.1. Study Design

**Figure 1:**
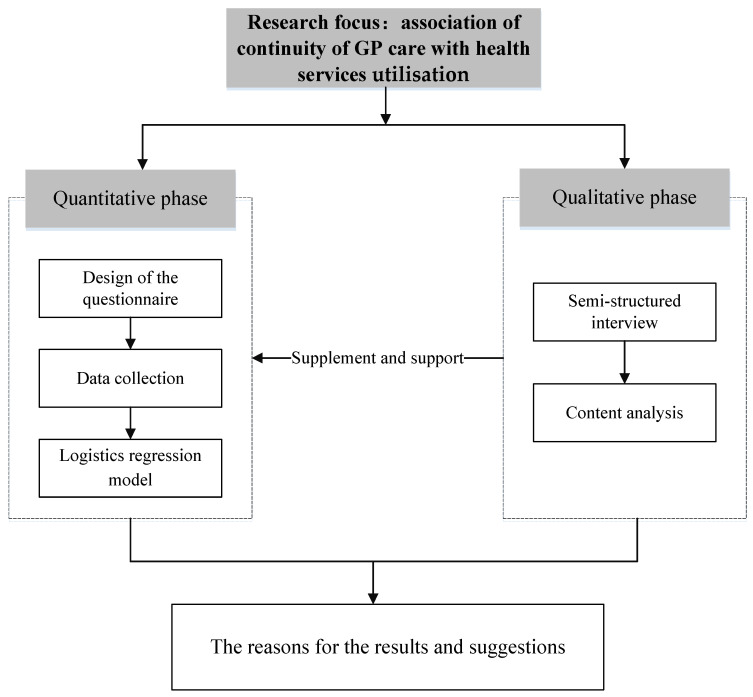
Process of mixed methods research.

We used a mixed methods approach in data collection and the analytical process. In the quantitative phase, a cross-sectional survey using a self-developed questionnaire was conducted on residents. Multiple regression models were established to analyse the association of continuity of GP care with health service utilisation. In the qualitative phase, the study was supplemented by an in-depth interview and content analysis to explore potential reasons for results in the quantitative phase. The process of mixed methods is shown in Figure 1 and described more fully below.

### 2.2. Quantitative Phase

#### 2.2.1. Data Collection and Subjects

We carried out a cross-sectional quantitative survey on residents in the urban community of Hangzhou, the capital city of an eastern developed province in China, from 1 to 31 July 2019. A multistage sampling strategy was adopted. In the first stage, five urban districts were selected based on location and level of economic development. In the second stage, we randomly selected two communities in each selected district. Lastly, two trained investigators were dispatched to each sampling community to randomly invite local residents to participate in our investigation [19]. Ethics approval was obtained from Hangzhou Normal University.

In this survey, the participants who were younger than 18 years old or lived in a sampling community for less than 6 months were excluded. Face-to-face interviews with residents were conducted, and participants were asked to read the informed consent letter and give oral consent before they filled out the questionnaires and volunteered to participate in this survey [20].

About 900 residents were invited to participate to this survey, and 858 (95.3%) participants were willing to fill in the questionnaire. The returned questionnaires containing missing values on the outcome and explanatory variables were excluded from data analyses, resulting in a final sample of 624. On the basis of the amount of independent variables included, this sample size was more than 20 times the amount of independent variables, which is large enough to estimate multiple regression models [21].

#### 2.2.2. Measurements

##### Outcome Variables

Three dependent variables were included in this study. The first one was intention to visit GP measured by the item in the questionnaire of ‘if you tend to visit GP firstly when feel discomfort’. The second was the frequency of GP visitations measured by ‘how many times have you visited GPs in the previous month because of discomfort (if not, please write zero)’. The last was the frequency of specialist visitations which was measured by the question ‘how many times have you visited specialists in the previous month because of discomfort (if not, please write zero)’.

##### Measurement of COC

Following Reid’s conceptual formwork [12], the synthetic COC can be classified into three types: IC, MC and RC. IC focuses on mutual doctor–patient knowledge; MC assesses the availability and flexibility of the doctor’s care; RC measures the ongoing patient–provider relationship. To measure the continuity of GP care in the Chinese primary healthcare system, a self-developed questionnaire was used to assess COC from these three dimensions consistent with previous studies [22,23]. This questionnaire included a total of 12 items (Table 1). IC was measured by five items, such as ‘I know the GP very well’. MC was measured by three items, such as ‘I feel comfortable consulting the GP about my doubts or health problems’. RC was measured by four items (for instance, ‘I can contact the GP easily when needed’). All items were rated according to a five-point Likert scale ranging from 1 (strongly disagree) to 5 (strongly agree).

To test the validity and reliability of this questionnaire, we adopted exploratory factor analysis (EFA) and confirmatory factor analysis (CFA), respectively. According to the results of EFA (varimax rotation), three common factors were extracted from 12 evaluation items, which fit the hypothesis model (Table S1). Cronbach’s α coefficients for these three common factors were higher than 0.8, indicating that the questionnaire had high reliability [24]. In addition, the structural equation model (SEM) was constructed for CFA. On the basis of the fit results, all fit indexes suggested that the model passed the goodness-of-fit test. For example, the root mean square error of approximation was 0.005; the goodness-of-fit index, comparative fit index and Tucker–Lewis index were higher than 0.9. All factor loading estimates in SEM were greater than 0.7 and *p*-values were less than 0.001, which indicated that our measurement tool was acceptable [25]. More details about EFA and CFA can be found in Supplementary Materials.

##### Other Covariates

To control the effect of other covariates on the association of COC with health service utilisation, some adjustment independent variables, such as gender, age, education, household monthly income per capita, marital status, medical insurance, chronic diseases and self-rated health status, were included in multiple regression models (Table 2) [26].

#### 2.2.3. Statistical Analysis

Before the data analysis, the scores of each item in the COC questionnaire were added, and an average score was calculated for IC, MC and RC. Each continuity index was transformed to a score ranging from 0 to 100. Finally, to simplify the analysis and the presentation of results, three types of continuity indexes were transformed into a categorical variable with two possible values: high versus low continuity [27]. The cut-off point for determining each type of continuity was developed based on the literature [28]. Values below the mean were coded as ‘low continuity’, whilst values above the mean were coded as ‘high continuity’ for further analysis.

In the data analysis stage, we firstly used Chi-square and nonparametric test to analyse the difference in intention of GP visitations, frequency of GP and specialist visitations in different levels of COC. To reduce estimations bias, bootstrap with 500 replications was adopted. The logistics regression model was established to assess the association between the level of COC and intention to visit GP. Simultaneously, we adopted Poisson regression to analyse the effect of level of COC on frequency of GP and specialist visitations. To control the influence of other factors, socio-economic characteristics and health status of respondents (gender, age, education, household monthly income per capita, marital status, medical insurance, chronic diseases and self-rated health status) were included in the above models as covariates. The significance level was set at 0.05. Statistical analyses were performed using the Statistical Package for Social Sciences version 20.0 (SPSS20.0).

### 2.3. Qualitative Phase

#### 2.3.1. Sampling and Interviews

With regard to the qualitative data collection, ample space was provided at the end of the structured part of the questionnaire to write the respondent’s comments regarding GP services. Sharing qualitative information was not compulsory for all respondents. Any respondent who was willing to share qualitative information was welcomed. As a result, a total of 26 respondents voluntarily accepted our interview. Before the interview, each interviewee was asked about their socio-demographics, followed by three questions: “How do you rate general practitioner services?”, “Why do you choose/not choose to seek the general practitioner when you get sick” and “Which aspects of general practitioner services are you most concerned about?” During the interview, one investigator was responsible for recording the interviewee’s comments, whilst the other was assigned to ask questions. The comments of respondents were written down on the spot and verbatim by the interviewer.

#### 2.3.2. Content Analysis

A thematic content analysis was used for qualitative study. Firstly, two researchers needed to read and check interview records prior to data analysis. Secondly, these interview materials were transcribed into text in Mandarin and imported into NVivo 7.0. This software is useful for qualitative analyses, enabling enhanced validity and rigor in qualitative analysis [29].

Using an inductive approach following principles of grounded theory [30], dominant themes were extracted from the comments considering the re-occurring categories and irregularities. Data reduction was performed manually. We classified the related comments into various categories. In this regard, working independently, two coders initially read the transcripts and broke the data down into codes, reread transcripts and identified and coded emerging categories [31]. The comments other than the scope of this study were not included in the analysis. The two sets of codes were compared using the kappa statistic to examine the inter-coder reliability for concordance [32]. Coding agreement between two raters was 94.5%. Disagreements were resolved through further discussion. In the next stage, the codes were later organised into themes and further into broader domains after adequate discussion. Finally, the theme-based variables were determined by consensus.

## 3. Results

### 3.1. Quantitative Findings

The characteristics of 624 subjects are displayed in Table 2. Females occupied more than half (60.1%) of the respondents. Approximately 44.6% of the respondents were aged between 26 and 35 years, and 42.0% of the respondents completed their bachelor’s degree. Monthly household income per capita varied considerably, with the monthly average income ranging from less than RMB 3000 (6.3%) to over RMB 10,000 (27.1%). Most of the respondents (71.6%) were married. Approximately 9.3% of the respondents were not covered by health insurance. The respondents with a fair or good self-rated health status comprised 46.8% and 45.4%, respectively. Moreover, 18.3% of the respondents reported they had at least one chronic disease.

With regard to the level of perceived COC, more than half of the respondents reported low IC (56.6%) and RC (57.7%) of GP services. However, 48.6% gave a high rating for MC.

Table 3 reports the distribution of different levels of COC in the visiting intention, frequency of GP and specialist visitations. Differences in the distribution of the different levels of IC, MC and RC on the intention to visit GP and frequency of GP visitation were statistically significant (*p* < 0.001). In general, those respondents who gave high scores on IC, MC or RC visited GP more frequently than their counterparts. Additionally, the results showed that the level of MC was negatively associated with frequency of specialist visitations (*p* < 0.05).

After controlling the characteristics of respondents (gender, ages, education, income, marital status, health insurance and health status), the results of multiple regressions were reported (Table 4). In terms of IC, those who reported higher scores on IC sought GPs firstly for treatment when they were sick (OR: 3.583; 95% IC: 1.828–7.024). Moreover, IC was found to be positively associated with the frequency of GP visitations (*β*: 0.418; 95% IC: 0.100–0.735). Regarding RC, the model showed a higher frequency of GP visitations for those giving a high rating on IC (β: 0.446; 95%CI: 0.089–0.804) than their counterparts. Furthermore, those people who experienced a good MC in GP service care could reduce frequency of specialist visitation (β: −0.438; 95% CI: −0.874 to −0.001).

In addition to the three types of COC, other socio-demographic variables were found to be associated with outcome variables. For example, age was observed to have a proportional relationship with intention to visit GP and GP visits. An increased income resulted in an increase in specialist visits. Those who were diagnosed with chronic disease and gave a poor rating on their health had a higher possibility to increase GP and specialist visitations than their counterparts.

### 3.2. Qualitative Findings

A total of 10 male and 16 female interviewees provided qualitative data. Most of them had a bachelor’s degree (61.5%) and were aged 40 or above (76.9%). Eighteen respondents suffered from chronic diseases.

After finishing coding, we identified three domains as reasons for interviewees visiting/not visiting GPs: service capabilities, doctor–patient interaction and time provision (Table 5). Indeed, most of the interviewees paid the most attention to the service ability of doctors when they needed medical services. In comparison with specialists in tertiary hospitals, GPs in CHCs had a relatively poor performance in terms of academic qualifications and experience. Accordingly, the interviewees believed that GPs cannot provide accurate diagnosis and effective treatment, especially for some complex diseases. By contrast, the quality of health services provided by specialists in tertiary hospitals was considered better than that by GPs.

Despite the advantages of specialists in diagnosis and treatment ability, some residents expressed that they also considered visiting GPs due to a good interaction between doctors and patients. For example, GPs can spend more time communicating with patients to fully understand their health condition during the consultation. Additionally, GPs are required to provide routine follow-up visits for patients with chronic diseases and give them guidance on diet and drug use. These unique advantages largely strengthened the connection and familiarity between GPs and patients. Another advantage mentioned by interviewees is the length of consultation with doctors. On the one hand, it only takes less than 15 min for the patients to walk to the nearest CHCs, and the waiting time for consultation is also much shorter than that of tertiary hospitals. On the other hand, GPs usually provide more meeting time, which improves patient-perceived quality of healthcare services. These characteristics of GP services reflect COC from different aspects and provide explanations for the association of continuity of GP care with patients’ behaviour on seeing doctors.

## 4. Discussion

The COC, widely known as the core value of high-quality patient care, has been proven to have a positive association with health outcomes [11]. This study focused on the association of continuity of GP care with the intention to visit GP, frequency of GP and specialist visitations in China. As COC is composed of IC, MC and RC, the component must be specified whenever the association between continuity and outcomes is discussed.

Our findings indicated that IC had a positive association with intention to visit GP and frequency of GP visitation. IC reflects mutual knowledge and understanding in the GP–patient relationship, as well as exchange and transfer of knowledge and information between doctors and patients, which is helpful to guide current care [11]. A high level of IC provides benefits to patients by reinforcing the accumulation of patient knowledge, improving interpersonal communication and increasing the likelihood that patients adhere to their doctor’s advice [33]. Naturally, those patients who experience better communication with GPs also develop trust on capacity and competency of GPs [34], and they prefer their own practitioner who is familiar with their unique medical condition and background [17]. Qualitative findings also revealed that doctor–patient familiarity was considered an important advantage of GP services. The respondents believe that visits in the setting of mutual knowledge may be both more efficient and rewarding. On the basis of our findings, some measures related to the accessibility of doctor–patient information should be highlighted to strengthen the competency of GPs and increase patient’s intention of choosing primary healthcare setting for treatments. For example, internet and information systems (such as electronic health records, online medical consultation, remote consultation and medical information inquiry) can be implemented so that patients’ information is easily accessible and available to all healthcare providers.

Similar to prior studies [35,36], RC appears to be associated with frequency of GP visitations positively after controlling the impact of health demand (chronic diseases and self-rated health status). An ongoing and close relationship with a patient allows the physician to acquire knowledge, not only about the patient’s medical problems, but also about his or her attitudes and values. Accordingly, a patient with a sustained relationship with a physician is likely to foster trust in the physician’s expertise and medical judgment and to ask the physician for advice before going to visit [14,17]. Strengths and values of RC actually create an environment of trust that may be conducive to improved patient adherence with agreed-upon treatment. The evidence supporting the association between RC and GP service utilisation is found from our qualitative findings. The majority of the respondents paid more attention to the doctor–patient interaction because consistent contact between patients and providers increased mutual knowledge so as to guide the doctor to provide a more appropriate treatment plan. Therefore, important implications from this finding should take strengthening follow-up contact and communication with patients as a vital issue of improving the service capabilities of GPs.

However, one GP team in China is required to offer public health services for over 1,000 residents in a local community. The severe shortage in qualified GPs resulted in the fact that primary healthcare providers have no time for routine follow up of patients, and communication between patients and providers is particularly limited; ultimately, a high-level RC in actual practice is very difficult to achieve, especially in the GP-based primary healthcare model [4,37]. Training more qualified GPs and reducing workload of GPs should be prioritised in the near future.

Consistent with other studies [20,38], our findings also showed that those respondents who gave a high rating for MC took the initiative to reduce specialist visitation. As mentioned above, consistency and flexibility of healthcare are identified as two core dimensions of MC, with the requirement that physicians should keep the planned care pathway coordinated, and adopted care should be changed in an individual’s needs and circumstances [39]. In actual practice, providers spend more time during visits and provide appropriate and affordable services to patients as much as possible. MC of primary care also emphasises the value of patient participation, which was consistent with previous findings that patients are more likely to adhere to treatment, and treatment outcomes can improve largely when the physician considers the patient an active participant in treatment [40]. By contrast, overtreatment and unsuitable services without considering patients’ actual needs are often provided by specialist care in China’s tertiary hospitals [41]. For example, patients with simple health problems are prescribed more drugs and asked to take unnecessary tests. As a result, patients have to bear a heavy financial burden arising from these unreasonable treatments. In such context, improved MC of GP care might reduce specialists’ visitations even though their strengths in capabilities in diagnosis and treatment still exist.

In accordance with our findings, non-technical or ‘soft’ quality of services in primary care setting should be especially highlighted in competition with tertiary hospitals in China. However, the training model for GPs is still consistent with that of specialists. GPs are taught more knowledge about diagnosis and treatment, rather than a comprehensive and health promotion-oriented approach [37]. Consequently, most GPs still hold a disease-centred belief instead of patients, resulting in unique advantages of GP services in mutual doctor–patient knowledge and communication that have not been exploited yet in China [4,37,42]. Most GPs reported that a lack of technique in behavioural medicine and psychological intervention is their main obstacle in their practice. In the long term, comprehensive primary healthcare models focusing on the person and their relational networks, adopting a life-long, promoting, preventive and curative vision, and taking into account the biological dimensions of the disease together with the individual social aspects should be taken into consideration for improving continuity of services in China [43].

This study has some limitations. Firstly, the limited sample size involved only Hangzhou City, so generalisation of the findings should be done cautiously. Secondly, this cross-sectional survey did not allow us to evaluate a cause–effect relationship between COC and health service utilisation. Given the adoption of self-reported questions about the frequency of GP and specialist visitations, there might be reporting bias in outcome variables, especially for the elderly.

## 5. Conclusions

Our study showed a strong association between continuity of GP care and healthcare service utilisation. Specifically, patients with high ratings of IC and RC tend to use GP services more frequently, but patients who experience a high MC might reduce specialist visitations. The findings imply that comprehensive health management capabilities of GPs should be prioritised when designing a GP system in China, especially in the use of health information, doctor–patient interaction and the provision of coordinated services.

## Figures and Tables

**Table 1:** Items for measuring continuity of GP care.

Dimension	Items
IC	1. I know the GP very well
	2. I believe that the GP knows my previous medical history very well
	3. I believe the GP knows my living environment very well
	4. I believe the GP knows my daily activities very well
	5. I believe the GP knows a general level of my health very well
MC	6. I feel comfortable consulting the GP about my doubts or health problems
	7. During the consultation process, the GP can inquire about my physical condition carefully
	8. The GP is very clear about what kind of treatment is most effective for me
RC	9. I can contact the GP easily when needed
	10. I go to visit the same GP every time I become sick
	11. The GP can provide services in my home, such as home visits or home care beds
	12. The GP can communicate with me and teach me health knowledge

**Table 2:** Characteristics of respondents.

Characteristics of Respondents	*n*	%
Gender		
Male	249	39.9
Female	375	60.1
Age (years)		
18–25	128	20.5
26–35	278	44.6
36–45	112	17.9
46–55	57	9.1
≥56	49	7.9
Education		
Primary school or below	24	3.8
Junior high school	57	9.1
High school	156	25.0
Bachelor’s degree	262	42.0
Graduate degree or above	125	20.0
Household income per capita (¥RMB)		
<3000	39	6.3
3000–	162	26.0
5000–	153	24.5
8000–	101	16.2
10,000≤	169	27.1
Marital status		
Single	138	22.1
Married	447	71.6
Other	39	6.3
Medical insurance		
Uninsured	58	9.3
Insured	566	90.7
Chronic diseases		
Yes	114	18.3
No	510	81.7
Self-rated health status		
Bad	49	7.8
Fair	292	46.8
Good	283	45.4
IC		
Low	353	56.6
High	271	43.4
MC		
Low	321	51.4
High	303	48.6
RC		
Low	360	57.7
High	264	42.3

**Table 3:** Differences in the distribution of different COC levels on intention to visit GP, frequency of GP and specialist visitations.

	Intention to Visit GP		GP Visits		Specialist Visits	
	No	Yes	Mean	SD *	Mean	SD
IC						
Low	258	95	0.60	1.08	0.59	0.90
High	142	129	0.68	1.15	0.63	1.09
*p*	<0.001		<0.001		0.488	
MC						
Low	240	81	0.79	1.37	0.64	1.02
High	160	143	1.15	1.56	0.49	0.83
*p*	<0.001		<0.001		0.036	
RC						
Low	266	94	0.60	0.95	0.55	0.98
High	124	130	1.32	1.25	0.45	0.73
*p*	<0.001		<0.001		0.099	

* SD, standard deviation.

**Table 4:** Prediction of COC for visiting intention, frequency of GP and specialist visitations.

Characteristics	Intention to Visit GP			GP Visits			Specialist Visits		
	OR *	95% CI *	*p*	β	95% CI	*p*	β	95% CI	*p*
IC (ref. = low)									
High	3.583	1.828–7.024	<0.001	0.418	0.100–0.735	0.010	0.128	−0.277–0.533	0.535
MC (ref. = low)									
High	0.924	0.436–1.959	0.837	−0.030	−0.412–0.352	0.878	−0.438	−0.874–−0.001	0.037
RC (ref. = low)									
High	1.758	0.843–3.666	0.132	0.446	0.089–0.804	0.014	−0.142	−0.637–0.243	0.381
Gender (ref. = male)									
Female	0.929	0.627–1.375	0.711	−0.053	−0.233–0.128	0.569	−0.009	−0.234–0.215	0.935
Age (ref. = 18–25)									
26–35	1.769	0.868–3.604	0.116	0.509	0.127–0.891	0.009	0.044	−0.401–0.490	0.846
36–45	2.666	1.171–6.161	0.020	0.541	0.106–0.975	0.015	0.224	−0.261–0.710	0.366
46–55	3.589	1.358–9.488	0.010	0.804	0.328–1.279	0.001	−0.106	−0.702–0.491	0.728
≥56	4.096	1.338–12.538	0.013	0.656	0.571–1.542	0.001	0.065	−0.562–0.692	0.839
Education (ref. = primary school or below)									
Junior high school	0.909	0.289–2.861	0.870	−0.236	−0.646–0.174	0.259	−0.410	−1.152–0.331	0.278
High school	0.627	0.212–1.849	0.397	−0.157	−0.551–0.237	0.434	0.342	−0.300–0.984	0.296
Bachelor’s degree	0.523	0.176–1.554	0.244	−0.152	−0.564–0.260	0.471	0.241	−0.416–0.898	0.472
Graduate degree or above	0.513	0.160–1.640	0.260	−0.386	−0.851–0.080	0.104	0.417	−0.268–1.102	0.233
Income (ref. ≤ 3000)									
3000–	0.362	0.162–0.809	0.013	0.664	0.233–1.095	0.003	0.759	0.090–1.428	0.026
5000–	0.297	0.131–0.676	0.004	0.544	0.087–1.001	0.020	0.543	−0.146–1.232	0.122
8000–	0.316	0.132–0.759	0.010	0.564	0.078–1.050	0.023	0.544	−0.167–1.255	0.134
≥10,000	0.154	0.065–0.367	0.000	0.561	0.095–1.027	0.018	0.940	0.256–1.625	0.007
Marital status (ref. = Single)									
Married	0.510	0.259–1.006	0.052	−0.022	−0.363–0.318	0.898	0.223	−0.179–0.333	0.295
Other	0.381	0.135–1.078	0.069	−0.405	−0.866–0.055	0.084	0.222	−0.307–0.461	0.446
Medical insurance (ref. = Uninsured)									
Insured	0.608	0.323–1.144	0.123	0.118	−0.231–0.466	0.509	0.406	−0.077–0.888	0.099
Chronic diseases (ref. = no)									
Yes	0.913	0.494–1.685	0.770	0.364	0.114–0.614	0.004	0.549	0.253–0.844	0.001
Self-rated health status (ref. = bad)									
Fair	0.807	0.376–1.732	0.582	−0.464	−0.733–−0.195	0.001	−0.330	−0.677–0.017	0.063
Good	0.953	0.439–2.071	0.903	−0.619	−0.905–0.333	0.000	−0.378	−0.739—0.016	0.041

* OR, odds ratio; CI, confidence interval.

**Table 5:** Reasons for interviewees visiting/not visiting GPs.

Domains	Associated Themes	Example of Verbatim Transcript
Service capabilities	Effective treatment (20 interviewees)	“…When I get sick, I go to a tertiary hospital, because the treatment is very effective, and the general practitioners in the community are not technical…” “…The diagnosis and treatment skills of general practitioners are not as good as those of specialists in tertiary hospitals, so I still choose tertiary hospitals…”
	Working experience and education of GPs (18 interviewees)	“…Most of the general practitioners’ academic qualifications are college or undergraduate qualifications, and the level of education is generally low…, but the qualifications of specialists in tertiary hospitals are mostly Ph.D.” “…Many general practitioners are very young and have no experience, so I have no confidence in their skills. I am afraid that there will be misdiagnosis and missed diagnosis…”
	Accuracy of diagnosis (16 interviewees)	“…Community health service centres lack a lot of medical equipment, and some laboratory tests cannot be done…, Only essential medicines are provided, and other medicines cannot be used…”
Doctor–patient interaction	Doctor–patient communication (19 interviewees)	“…For some minor illnesses, I usually visit a GP because he has time to communicate with me and tell me what I should pay attention to in my daily life…”
	Doctor–patient familiarity (17 interviewees)	“…I signed a GP as my family doctor. He often visits for follow-up visits, so he knows my situation well. So, it’s very convenient for me to visit GP…”
	Doctor–patient trust (14 interviewees)	“…I am a hypertensive patient and often come to community health service centres. I know general practitioners very well. They will prescribe different drugs for me according to my blood pressure. Currently my blood pressure is controlled very well, I trust them very much… Sometimes they followed up regularly and give some guidance on diet and drug use”
Time provision	Waiting time (15 interviewees)	“…The community health service centre is very close to my home. It is very convenient for me to come here to see a general practitioner. At the same time, it’s not like a tertiary hospital where you have to wait in line for a long time…”
	Service time (12 interviewees)	“…General practitioners also provide me with service lasting at least 15 min of and ask me carefully about my condition, but specialists in tertiary hospitals spend about 5 min each time…”

## Data Availability

The data sets analysed during this study are available from the corresponding author on reasonable request.

## Supplementary Materials

The following are available online at https://www.mdpi.com/article/10.3390/healthcare9091206/s1, Table S1: Factor loadings of items and Cronbach’s α of the three dimensions based on exploratory factor analysis; Table S2: Factor loading estimates based on confirmatory factor analysis; Figure S1: Structural equation model for confirmatory factor analysis.
