# Supplementary material for: Association of Continuity of General Practitioner Care with Utilisation of General Practitioner and Specialist Services in China: A Mixed-Method Study
Source: Healthcare (Basel). 2021 Sep 13;9(9):1206. doi: 10.3390/healthcare9091206 (PMC8465206; doi:10.3390/healthcare9091206)
Supplement: Supplementary file 1 [file healthcare-09-01206-s001.zip › healthcare-1312068-supplementary.pdf]

Supplementary Materials

Table S1. Factor loadings of items and Cronbach's α of the three dimensions using exploratory factor analysis

Questionnaire items	IC	MC	RC
1. I know the GP very well	0.780		
2. I believe that the GP knows my previous medical history very well	0.891		
3. I believe the GP knows my living environment very well	0.842		
4. I believe the GP knows my daily activities very well	0.853		
5. I believe the GP knows a general level of my health very well	0.673		
6. I feel comfortable consulting the GP about my doubts or health problems		0.728	
7. During the consultation process, the GP can inquire about my physical condition carefully		0.788	
8. The GP is very clear about what kind of treatment is most effective for me		0.719	
9. I can contact the GP easily when needed			0.749
10. I go to visit the same GP every time I become sick			0.762
11. The GP can provide services in my home, such as home visits or home care beds			0.839
12. GP can communicate with me and teach me health knowledge			0.846
Cronbach's α	0.897	0.835	0.912

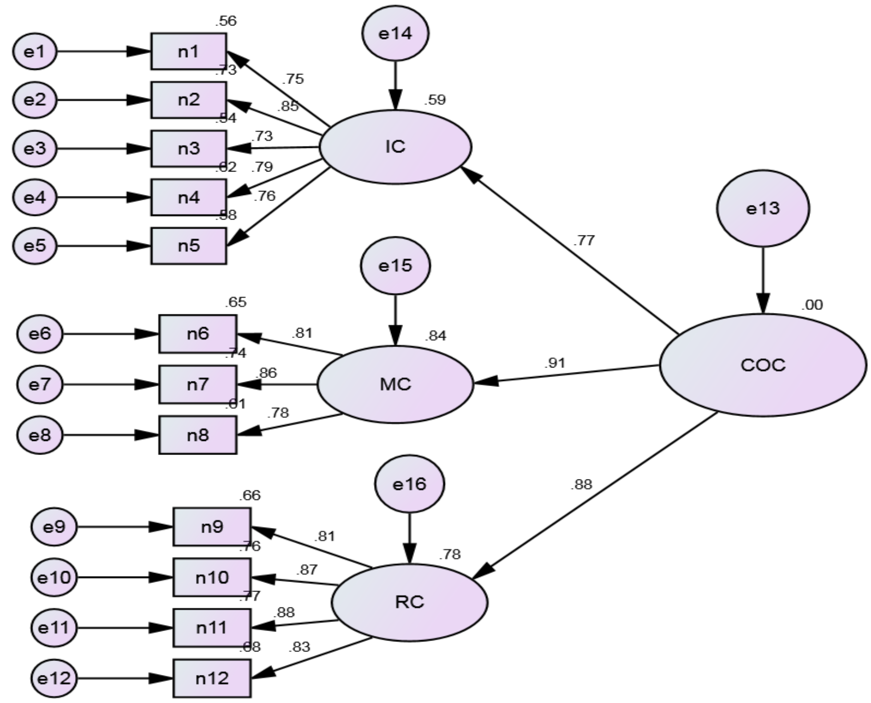

Figure S1 Structural equation model for confirmatory factor analysis

Table S2 Factor loading estimates using confirmatory factor analysis

Model paths			Coefficients
IC	←	COC	0.766^{**}
MC	←	COC	0.915^{**}
RC	←	COC	0.881^{**}
IC	←	N1	0.748^{**}
IC	←	N2	0.855^{**}
IC	←	N3	0.734^{**}
IC	←	N4	0.789^{**}
IC	←	N5	0.760^{**}
MC	←	N6	0.808^{**}
MC	←	N7	0.863^{**}
MC	←	N8	0.781^{**}
RC	←	N9	0.815^{**}
RC	←	N10	0.870^{**}
RC	←	N11	0.876^{**}
RC	←	N12	0.826^{**}

Note: **:$p<0.001$
